# Variation in the observed effect of Xpert MTB/RIF testing for tuberculosis on mortality: A systematic review and analysis of trial design considerations

**DOI:** 10.12688/wellcomeopenres.15412.2

**Published:** 2020-08-17

**Authors:** Eleanor A. Ochodo, Nelson Kalema, Samuel Schumacher, Karen Steingart, Taryn Young, Susan Mallett, Jon Deeks, Frank Cobelens, Patrick M. Bossuyt, Mark P. Nicol, Adithya Cattamanchi

**Affiliations:** 1 Department of Global Health, Stellenbosch University, Cape Town, Western Cape, 8000, South Africa; 2 Infectious Diseases Institute, Makerere University, Kampala, 22418, Uganda; 3 Tuberculosis Department, Foundation for Innovative New Diagnostics, Geneva, 1202, Switzerland; 4 Department of Clinical Sciences, Liverpool School of Tropical Medicine, Liverpool, L3 5QA, UK; 5 NIHR Birmingham Biomedical Research Centre, University Hospitals Birmingham NHS Trust, University of Birmingham, Edgbaston, Birmingham, UK; 6 Test Evaluation Research Group, Institute of Applied Health Research, University of Birmingham, Edgbaston, Birmingham, UK; 7 Amsterdam Institute for Global Health and Development, Amsterdam University Medical Centers, Amsterdam, 1105 BP, The Netherlands; 8 Deapartment of Clinical Epidemiology, Biostatistics and Bioinformatics, Amsterdam University Medical Centers, Amsterdam, 1105 AZ, The Netherlands; 9 School of Biomedical Sciences, Faculty of Health and Medical Sciences, University of Western Australia, Perth, WA, 6009, Australia; 10 Division of Pulmonary and Critical Care Medicine, University of California San Francisco Medical Center, San Francisco, California, 94110, USA

**Keywords:** Tuberculosis diagnosis, methodology, Diagnostic trials, Impact studies

## Abstract

**Background: **Most studies evaluating the effect of Xpert MTB/RIF testing for tuberculosis (TB) concluded that it did not reduce overall mortality compared to usual care. We conducted a systematic review to assess whether key study design and execution features contributed to earlier identification of patients with TB and decreased pre-treatment loss to follow-up, thereby reducing the potential impact of Xpert MTB/RIF testing.

**Methods: **We searched the Cochrane Central Register of Controlled Trials (CENTRAL), MEDLINE, and Scopus for literature published from 1 ^st^ January 2009 to February 2019. We included all primary intervention studies that had evaluated the effect of Xpert MTB/RIF on mortality compared to usual care in participants with presumptive pulmonary TB. We critically reviewed features of included studies across: Study setting and context, Study population, Participant recruitment and enrolment, Study procedures, and Study follow-up.

**Results: **We included seven randomised and one non-randomised study.  All included studies demonstrated relative reductions in overall mortality in the Xpert MTB/RIF arm ranging from 6% to 40%. However, mortality reduction was reported to be statistically significant in two studies. Study features that could explain the lack of observed effect on mortality included: the higher quality of care at study sites; inclusion of patients with a higher pre-test probability of TB leading to higher than expected empirical rates; performance of additional diagnostic testing not done in usual care leading to increased TB diagnosis or empiric treatment initiation; the recruitment of participants likely to return for follow-up; and involvement of study staff in ensuring adherence with care and follow-up.

**Conclusion: **Most studies of Xpert MTB/RIF were designed and conducted in a manner that resulted in more patients being diagnosed and treated for TB, minimising the potential difference in mortality Xpert MTB/RIF testing could have achieved compared to usual care.

## Introduction

Tuberculosis (TB) is the leading cause of mortality from an infectious disease globally. The 2018 World Health Organization (WHO) TB report estimates that there were 10 million incident TB cases and about 1.6 million TB-related deaths in 2017 ^1^. Early TB case detection and treatment initiation are critical for TB care and global TB elimination.

Sputum smear microscopy remains the primary method for diagnosing pulmonary TB in most countries with a high TB burden. Microscopy has suboptimal sensitivity and requires patients to submit multiple sputum samples often over several days, leading to loss to follow-up and missed opportunities for case detection and treatment. Nucleic acid amplification tests (NAAT) are known to increase sensitivity but until recently were not feasible in high-burden countries ^2^. In 2010 ^3^, WHO first recommended Xpert MTB/RIF (Cepheid, Sunnyvale, CA, USA), a semi-automated, cartridge-based NAAT, as a first-line TB test for all patients suspected to have multi-drug resistant TB or HIV-associated TB and in 2013 ^4^, revised the recommendation to include Xpert MTB/RIF testing for all patients suspected to have TB where resources permit.

Since the initial WHO recommendations based on diagnostic accuracy estimates, several trials ^5– 12^ have evaluated whether Xpert MTB/RIF testing reduced mortality among those undergoing TB evaluation in comparison to smear microscopy or pre-existing diagnostic algorithms. These trials have reported variable estimates of reduction in mortality, with only two ^9, 11^ reporting a statistically significant decrease in mortality. A recently published individual patient data meta-analysis of five of such trials ^6– 8, 10, 13^ also did not show significantly reduced six-month all-cause mortality (OR 0.88, 95% CI 0.68 to 1.14) in adults ≥18 years with presumptive pulmonary TB ^14^.

Available literature cites possible reasons to explain methodological limitations of test-treatment trials and Xpert MTB/RIF’s apparent lack of significant effect on mortality. A methodological review of test-treatment trials (n=103) published between 2004 and 2007 concluded that such trials were probably underpowered and had issues related to blinding, attrition, and inadequate primary analyses ^15^. Other reviews of trials of Xpert MTB/RIF have raised issues related to the health systems in which the trials were conducted ^16^, limited study power ^14, 16^, persistent use of empirical therapy ^17^, limitations in interpreting trial results by focusing on statistical significance rather than clinically important differences ^18^, enrolling patients whose test results are not likely to influence treatment decisions or limitations in evaluating a diagnostic test itself rather than a diagnostic test strategy in the intervention arm ^19^. However, to date, less attention has been paid to the external validity of trials: the extent to which the design and conduct of the trials reflect what could be expected in usual care. In addition to earlier identification of drug resistance, Xpert MTB/RIF testing is expected to reduce mortality through earlier identification of patients with TB (increased sensitivity compared with smear microscopy) and decreased pre-treatment loss to follow-up (faster turn-around-time for results). We conducted a systematic review to assess whether the design and/or execution of studies also contributed to earlier identification of patients with TB and decreased pre-treatment loss to follow-up, thereby reducing the potential impact of Xpert MTB/RIF testing.

## Methods

### Study identification

We conducted a literature search to identify randomised and non-randomised studies assessing mortality following the introduction of Xpert MTB/RIF testing. We searched the Cochrane Central Register of Controlled Trials (CENTRAL), MEDLINE, and Scopus for studies in English published between 1 January 2009 and February 2019 with the terms ‘Xpert MTB/RIF’ or ‘Xpert’ or ‘GeneXpert’ and ‘impact’ or ‘effect*’ or ‘implementation’ or ‘trial*’. We included studies that compared Xpert MTB/RIF to usual care as defined by the authors (for example sputum microscopy or culture), intending to measure the effect of these tests on mortality among participants presumed to have active pulmonary TB. Hypothetical trials or modelling studies were excluded. The study protocol, details of which are available as *Extended data* ^20^, followed PRISMA guidelines for performing systematic reviews, where applicable ^21, 22^; however, since this was not a classical systematic review, not all items were appropriate. A completed checklist is available from Open Science Framework ^20^.

### Appraisal of studies

One reviewer (NK) searched, identified and appraised eligible articles up to December 2016. A second reviewer (EO) updated the search, identified and appraised eligible articles up to February 2019 in discussion with a senior reviewer (AC). The study data were extracted using Google forms and included the following elements: general study characteristics (geographical location, TB and HIV co-infection); description of study arms; sample size and power; description and results of the mortality outcome; and description of key study design features (study setting and context; study population; participant recruitment and enrolment; study procedures and participant follow-up). We used descriptive statistics to summarise quantitative data and provide a narrative summary of key design features concerning their potential impact on usual care. In appraising usual care, we considered how the study was executed assessing if usual care was enhanced beyond what is considered routine ^23– 27^.

## Results

### Characteristics of included studies

**Figure 1.**
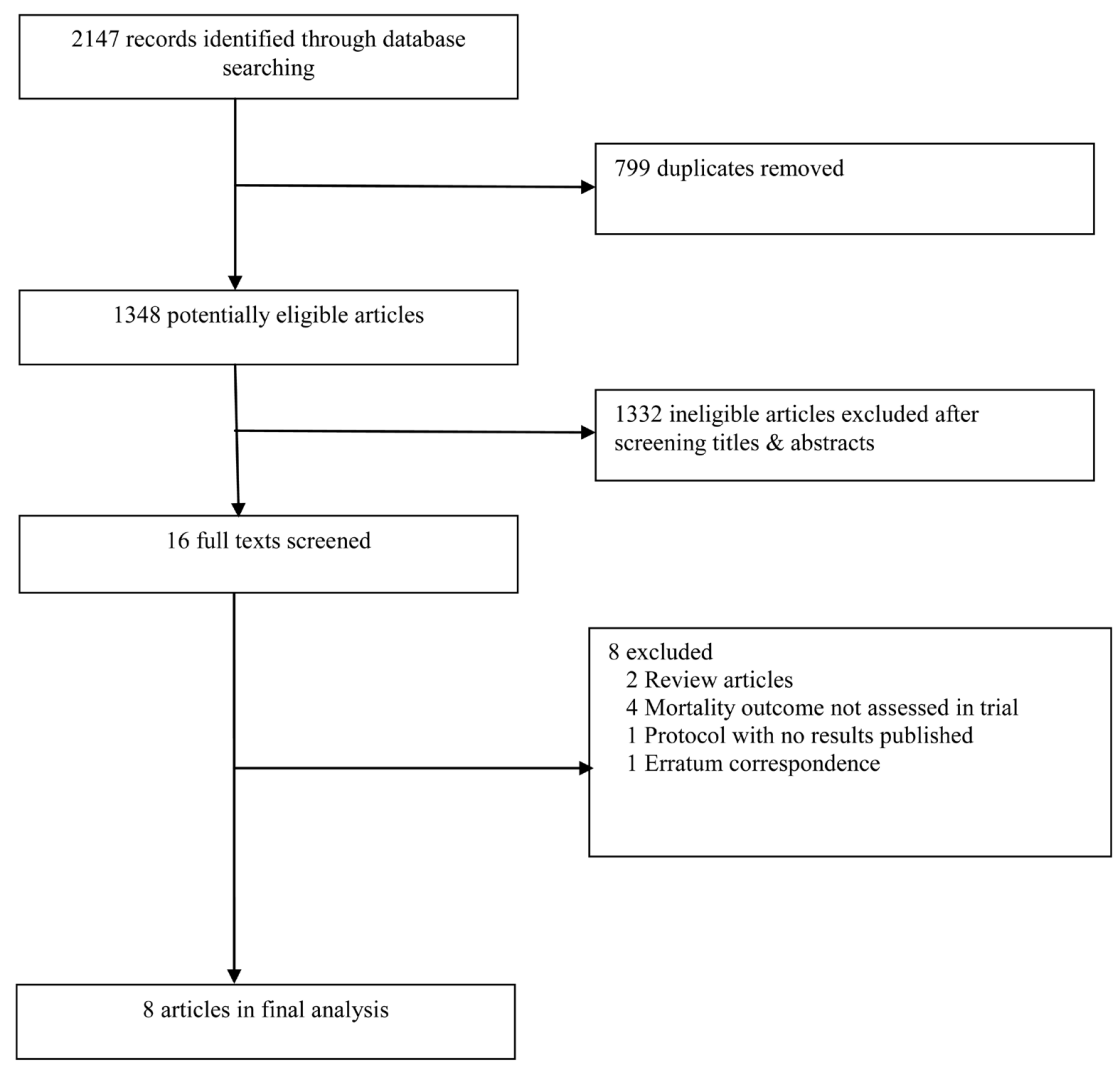
Flow chart of included studies.

Our search yielded 2147 records ( Figure 1). From this, eight studies were included in this review ( Table 1) ^12^. These studies comprised three individual randomized trials ^5, 8, 10^, two cluster randomised trials ^6, 9^, one secondary analysis of a stepped wedged randomised trial ^11, 13^, one cross-over trial ^7^, and one pre-post intervention study ^12^. Further information about each trial is given as *Extended data* ^20^.

**Table 1.** Table of included study characteristics.

	Design and setting	Study population	Study arms	Rate of empirical treatment	Loss to follow up	Mortality outcome
Calligaro *et al.* (2015) ^5^ South Africa	Two arm individual randomised trial nested within a prospective cohort study done at ICUs of four tertiary referral centres in Cape Town.	Mechanically ventilated adults (≥18 years) with suspected pulmonary TB (In-patients) HIV+ status (27%)	Intervention: Xpert MTB/ RIF (N=111) Control: Smear microscopy on tracheal aspirates using LED Fluorescence microscopy and culture (N=115)	Xpert:n=4/24 * (17%) Smear:n=9/16 *(56%) *Among* *Patients started on antiTB* *treatment* 13/40=32.5%	Overall N=11/317=3.5% Xpert:NR Smear: NR	Mortality-secondary outcome 28-day mortality: 30/111 (27%) vs 39/115 (34%); *RR=0.80[95%CI 0.54-1.19] 90-day mortality: 36/111 (32%) vs. 48/115 (42%); *RR=0.78(95%CI 0.55-1.10)
Churchyard *et al.* (2015) ^6^ South Africa	Two-arm parallel cluster-randomised trial at primary care clinics in medium- burden districts of South Africa	Adults (≥18 years) with suspected pulmonary TB (Outpatients) HIV+ status (62%)	Intervention: Xpert MTB/ RIF on one sputum sample in associated laboratory N=10 laboratories N=2324 individuals Control: Smear microscopy on 2 sputum samples using LED Fluorescence microscopy N=10 laboratories N=2332 individuals	Xpert: NR Smear:NR	Overall N=48 (1%) Xpert: n=25 Smear: n=23 Initial LTFU among those with positive index test: N=60/374 (16%) Xpert: n= 34/200(17%) Smear: n=26/174(14.9%)	Six-month mortality-primary outcome 91/2324 (3.9%) vs. 116/2332 (5%) †Unadjusted HR 0·86 (95% CI 0·56– 1·28) †aHR 1.10 (95%CI 0.75- 1.62)
Cox *et al.* (2014) ^7^ South Africa	Two-arm cross-over trial conducted in one large primary health care clinic in a peri-urban township in Cape Town. Randomly allocation as either Xpert or routine diagnostic testing occurred each week.	Adults (≥18 years) with suspected pulmonary TB (Outpatients) HIV+ status (47%)	Intervention: Xpert MTB/RIF on two sputum sample in onsite laboratory: N=982; Control: Routine diagnostic testing on two sputum samples in onsite laboratory: N=1003;	Xpert:NR Smear: NR Reported *“In our study* *population, empiric treatment is* *less common”*	Xpert:NR Smear: NR	Six-month mortality- secondary outcome 3.4% (33/982) vs.3.8% (38/1,003) ¥RR=0.89, [95% CI 0.56–1.40]
Mupfumi *et al.* (2014) ^8^ Zimbabwe	Individual randomised clinical trial at one ART initiation center in an urban setting	HIV-infected adults (≥18 years) initiating ART (Outpatients) HIV+ status (100%)	Intervention: Xpert MTB/RIF on one sputum sample (N=182) Control: Same-day Smear microscopy on two sputum samples using LED Fluorescence microscopy (N=172)	Among those diagnosed with TB by bacteriological or clinical criteria at baseline Overall N=54/88=61% Xpert: n=23/43 (54%) Smear: n=31/45 (69%)	Overall N=60 (17%) Xpert: n=32/182 (18%) Smear: n=38/172 (22%)	Three-month mortality- primary outcome 11/182(6%) vs. 17/172(10%) *RR=0.61 [95%CI 0.29-1.27]
Ngwira *et al.* (2018) ^9^ Malawi	Two-arm cluster- randomised trial at 12 rural primary health clinics	Adults (≥18 years) newly diagnosed with HIV with suspected pulmonary TB. Not on ART (Out-patients) HIV+ status (100%)	Intervention: Point-of-care Xpert MTB/RIF on one sputum sample (onsite same day) (N=1001) Control: Point-of-care Smear microscopy on two spot sputum samples using LED Fluorescence microscopy (onsite same day) (N=841)	Xpert: NR *Reported that more clinical* *diagnoses observed in the Xpert* *arm than in the LED FM arm but* *figures not reported*. Smear: NR	Overall N=407 (22%) Xpert; n=220 (22%) Smear; n=187 (22%)	12-month mortality- primary outcome †6.7 vs. 8.6 per 100 person- years RR=0.78[95% CI: 0.58-1.06] *Sub group analysis* (Advanced HIV) (RR 0.43, 95%CI: 0.22-0.87).
Theron *et al.* (2014) ^10^ South Africa, Zimbabwe, Zambia, Tanzania	Individual randomised clinical trial at peri- urban primary health care TB clinics	Adults (≥18 years) with suspected pulmonary TB (Outpatients) HIV+ status (60%)	Intervention: Onsite, nurse- done Xpert testing on one sputum sample (N=744) Control: Same-day smear microscopy on one sputum sample (N=758)	Xpert: n=130/744 (17%) Smear:n= 197/758 (26%)	Overall N=20% Two months Xpert: n=69/321 (21%) Smear: n=70/324 (21%) Six months Xpert: n=74/321 (23%) Smear: n=71/324 (22%)	Six-month mortality- secondary outcome 58/744(8%) vs.63/758 (8%) †RR=0.94[95%CI 0.67-1.32]
Trajman *et al.* (2015) ^11, 13^ Brazil	Secondary analysis of a stepped wedge cluster randomised trial. Trial was conducted in public health primary care clinics	Patients (0 to ≥60 years) notified with pulmonary TB in the Brazilian national TB information system HIV+ status (10%)	Intervention: Xpert MTB/ RIF on one sputum sample in laboratory (N=2232) Control: Smear microscopy using conventional light microscopy based on direct Ziehl-Neelsen staining on two sputum samples in laboratory (N=1856)	Xpert: NR -Clinically diagnosed, negative test n= 332 (14.9%) -Clinically diagnosed, no test result n=199 (8.9%) Smear:NR -Clinically diagnosed, negative test n= 381 (20.5%) -Clinically diagnosed, no test result n=213 (11.5%) Overall empirical treatment: 27.5%	Overall N=656 (16%) Xpert; n= 356 (15.9%] Smear; n= 300 (16.2%]	15 to 23-month mortality- secondary outcome 52/2232 (2.3%) vs. 71/1856 (3.8%) *OR=0.60[95% CI 0.42-0.86] †aOR (HIV status and age group)=0.65, 95% CI=0.44- 0.97
Yoon *et al.* (2012) ^12^ Uganda	Pre- and post- implementation study at a national referral hospital in capital city	Adults (≥18 years) with suspected pulmonary TB (In-patients) HIV+ status (76%)	Intervention: Xpert MTB/ RIF on one sputum sample (N=190) Control: Smear microscopy on two sputum samples using fluorescence microscopy (N=287)	Among those with culture- confirmed TB (n=262; 12%) Xpert: 7/105 (7%) Smear:24/157 (15%)	Overall N=32 (6%) Xpert: n=4 (2%) Smear: n=28 (10%)	Two-month mortality- Primary outcome †14% vs. 17% (Among 252 bacteriologically confirmed TB difference +3%, 95% CI: -21% to +27%,) *Overall mortality 55/250 vs 35/177 *RR=0.90[95%CI 0.62-1.31]

Abbreviations: NR, Not reported; ART, Anti-retroviral therapy *Estimates Relative Risk and 95% confidence intervals calculated by review team from mortality estimates presented in study. † Estimates reported in included studies

Each study was described as pragmatic by the study authors and involved patients undergoing evaluation for pulmonary TB in routine care settings (primary health care clinics ^6– 11^ and tertiary referral hospitals ^5, 12^). All eight studies were conducted in high-TB-burden countries ^28^, including seven in sub-Saharan Africa ^5– 10, 12^, and one in Brazil ^11^. Seven studies included adults ≥18 years ^5– 10, 12^ and one study ^11^ included adults and children of any age. Proportion of HIV-positive participants in the included studies ranged from 10% to 100%.

Usual care consisted of sputum smear microscopy in all but one study, where both culture and smear microscopy comprised standard of care ^5^ following a change in government policy recommending Xpert MTB/RIF as the initial diagnostic test.

Overall rates of participant loss to follow-up (LTFU) ranged from 1% to 22% in included studies. LTFU rates between trial arms were similar except for two studies in which LTFU was higher in the smear microscopy arm compared to the Xpert MTB/RIF arm (10% vs 2% ^12^ and 22% vs 18% ^8^, respectively).

All-cause mortality was evaluated in seven studies ^5– 9, 12, 17^ and TB-attributed mortality in one study ^11^. Mortality was assessed as the primary outcome in three studies ^6, 9, 12^, as a composite primary outcome in one study ^8^ and as a secondary outcome in the other four studies ^5, 7, 11, 17^.

All included studies demonstrated relative reductions in overall mortality in the Xpert MTB/RIF arm ranging from 6% to 40%. However, mortality reduction was reported to be statistically significant in two studies ( Table 1) ^9, 11^. Ngwira and colleagues ^9^ reported a statistically significant reduction in all-cause mortality in a subgroup of patients with newly diagnosed advanced HIV at primary health clinics in Malawi (RR: 0.43, 95% CI: 0.22 to 0.87), but not in the overall study population (RR: 0.78, 95% CI: 0.58 to 1.06). Trajman and colleagues ^11^ reported a lower TB-attributed death rate in the Xpert MTB/RIF arm (2.3% vs 3.8%; OR: 0.60, 95% CI: 0.42 to 0.86) among patients with presumptive TB in primary health clinics in Brazil.

## Analysis of key study design features relative to usual care

We analysed study features across five domains: study setting and context, study population, participant recruitment and enrolment, study procedures, and study follow-up. A summary of study features can be found in Table 2.

**Table 2.** Analysis of study features relative to usual care.

Study	Summary of study features
Calligaro *et al.* (2015) ^5^	Used laboratories that observed high quality standards for TB testing Inpatient setting with high pretest probability of TB and empirical treatment Informed consent required Use of additional testing; culture
Churchyard *et al.* (2015) ^6^	High quality laboratories used; laboratories not meeting standards excluded Patients with reduced chance of LTFU. Excluded patients from remote locations or from outside catchment area Informed consent required Enhanced follow up by giving airtime vouchers to maintain contact; Home visits to those who lost contact
Cox *et al.* (2014) ^7^	Used laboratories that observed high quality standards for TB testing Informed consent waived Enhanced follow-up of patients using multiple existing data registries used to follow-up patients; Home visits made to test negative patients and test positive patients not on treatment
Mupfumi *et al.* (2014) ^8^	Used laboratories that observed high quality standards for TB testing Population with high likelihood of empirical treatment; HIV positive patients starting ART Informed consent required Use of additional testing; chest radiographs Enhanced follow up by tracking LTFU through clinical records and home visits
Ngwira *et al.* (2018) ^9^	Used laboratories that observed high quality standards for TB testing Population with high likelihood of empirical treatment; HIV positive patients starting ART Patients with minimal chance of LTFU. Excluded patients from remote locations or from outside catchment area Informed consent required Continuous training of onsite personnel Enhanced follow-up of patients though extra visits, home visits and using data registers
Theron *et al.* (2014) ^10^	Used laboratories that observed high quality standards for TB testing Research staff directly involved in care of participants Higher pre-test probability of TB; HIV negative patients required to have ≥ 2 symptoms of TB Informed consent required Likelihood of increased interaction between study staff and patients through transporting patients for additional testing and counselling Use of additional testing; chest radiographs and culture
Trajman *et al.* (2015) ^11, 13^	Used laboratories that observed high quality standards for TB testing Informed consent waived All patients with presumptive TB included; no exclusion criteria Utilised routinely collected data from electronic records database No additional staff and diagnostics relative to usual care No enhanced follow up strategies
Yoon *et al.* (2012) ^12^	Used laboratories that observed high quality standards for TB testing Research staff directly involved in care of participants Research staff performed sputum induction and bronchoscopy Inpatient setting with high pretest probability of TB and empirical treatment Informed consent required Likelihood of increased interaction between study staff and patients through transporting patients for additional testing and counselling Use of additional testing; chest radiographs and culture Enhanced follow up by providing transport vouchers and home visits

### Study setting and context

We focused on whether the quality of care in the usual care arm was higher at study sites than would be expected in usual care settings, either because of the sites chosen or the manner in which studies were executed. All eight studies used laboratories that observed high quality standards for TB testing, with one study ^6^ excluding laboratories that did not meet quality standards. In two studies, research staff were directly involved in the care of participants, including facilitating chest X-rays, delivering test results to participants, and referring participants for TB treatment ^10, 12^. In one study, research staff performed sputum induction and bronchoscopy, neither of which were routinely available at the study site ^12^.

### Study population

Empiric treatment is more common when pre-test probability of TB is high ^17, 19^ or in study populations with very ill patients who have a high likelihood of dying, reducing the potential impact of Xpert MTB/RIF, a more sensitive test than sputum microscopy. Five studies reported rates of empiric treatment, and the rates ranged from 12% to 60% ^5, 8, 10– 12^. Five of eight studies enrolled participants with a higher pre-test probability of TB than the target population (i.e., ** all patients referred for sputum-based TB testing in usual care). Yoon and colleagues ^12^ and Calligaro and colleagues ^5^ conducted their studies in inpatient settings, where TB prevalence and empiric treatment rates are generally higher than in outpatient settings. Theron and colleagues ^10^ required HIV-negative participants to have at least two TB symptoms (cough for more than two weeks, fever lasting two weeks, weight loss, sweats, fatigue, chest pain or hemoptysis), rather than enrolling all patients referred for TB testing. Two studies ^8, 9^ included only HIV-positive patients who had not started ART, a population in whom empiric treatment is more common. In addition to high rates of empiric treatment, Churchyard and colleagues ^6^ and Ngwira and colleagues ^9^ excluded patients who resided outside the clinic catchment area or in remote locations, reducing the potential for loss to follow-up.

### Participant recruitment/enrolment

A high level of interaction between research staff and participants could lead to increased adherence to care and follow-up. Study staff requested consent from participants in all but two ^7, 11^ studies, and as noted earlier, transported patients for chest radiographs in two studies ^10, 12^. Both studies provided an opportunity for research staff to build rapport and counsel and educate participants on TB diagnosis and treatment. In addition, patients were asked to wait for their smear microscopy results or were offered voluntary counselling as they were being transported for chest radiographs in one study ^10^, likely reducing pre-treatment loss to follow-up relative to routine care. 

### Study procedures

The use of testing and other procedures not typically available in many high burden settings could lead to more patients being diagnosed with and treated for TB than would have occurred under usual care. For example, chest radiography was performed in all participants in two studies ^10, 12^, at baseline at the discretion of clinicians in one study ^8^. The availability of chest radiograph results compatible with active TB is likely to have made empiric TB treatment initiation more frequent, especially for HIV-positive participants ^10^. Culture is not routinely available in most high TB burden settings. However, it was part of usual care in one study setting ^5^, and was performed in two other studies as a reference standard for diagnostic test accuracy calculations ^10, 12^. In all these three studies, a positive culture test result also informed treatment in the Xpert MTB/RIF arm.

### Study follow-up

To maintain contact and study follow up, Churchyard and colleagues ^6^ sent mobile phone call vouchers (worth $2 USD) as an incentive to encourage patients to remain contactable during the study and later organised home visits when contact calling failed. Ngwira and colleagues ^9^ enhanced follow up by scheduling extra visits, conducting home visits and using data registers to trace participants who missed clinic appointments. Yoon and colleagues provided transport vouchers and made home visits for patients who did not return for scheduled follow-up visits ^12^. The enhanced follow-up procedures likely increased initiation of TB treatment for those with bacteriologically-confirmed disease and those without bacteriological confirmation but persistent symptoms.

## Discussion

Our review has implications for the design of future trials aiming to assess the comparative effectiveness of novel TB diagnostics. We highlight features of trial design and execution that could have mitigated the key advantages of Xpert MTB/RIF relative to smear microscopy with respect to faster diagnosis and treatment of TB patients. Such features included: a higher quality of care in comparison to usual care at trial sites, inclusion of patients with higher pre-test probability of TB relative to all patients undergoing TB testing at the trial sites leading to higher than expected empiric treatment rates, selection criteria and increased contact with participants as a result of study procedures leading to reduced pre-treatment loss to follow-up, the performance of additional diagnostic testing not done in usual care leading to increased TB diagnosis or empiric treatment initiation, the recruitment of participants likely to return for follow-up, and involvement of study staff in ensuring adherence with care. Designing future comparative studies of novel TB diagnostics in real life settings where optimal conditions are not likely to be met could mitigate these issues and provide a better assessment of their likely impact.

Our findings complement those of Auld and colleagues ^16^, who also published a literature review exploring Xpert MTB/RIF’s lack of effect on morbidity and mortality. They appraised eight trials (six randomised ^5– 8, 10, 11^ and two pre-post trials ^12, 29^) and concluded that study characteristics that may explain this lack of effect on morbidity and mortality include underpowered trials, higher rates of empiric treatment in the control arms compared to the Xpert MTB/RIF arm, studies with populations not comprised exclusively of those likely to benefit from Xpert MTB/RIF, and health system limitations such as patient loss to follow-up. Our review extends upon and contextualizes these findings by focusing on how specific study design and execution features that improve upon usual care may mitigate the potential benefit of novel diagnostics.

Of the eight studies included in our review, Trajman and colleagues ^11^ minimally interrupted usual care for that setting. The study was conducted at public primary care settings, included all patients undergoing TB testing (no exclusion criteria) and utilized routinely collected data to assess outcomes. Electronic records of routinely collected diagnostic, treatment and outcome data were linked and analysed retrospectively with minimal influence by external research staff. Trajman and colleagues also did not utilize additional resources in terms of staff or diagnostics that were used over and above what was available in usual care settings and similar implementation protocols were uniformly applied at all sites. Informed consent was also not a requirement.

There is an inherent tension between a study’s internal and external validity ^30, 31^, with the former favouring more rigorous control and the latter more pragmatism. Indeed, most research on which practice guidelines have been based have focused on internal validity rather than external validity ^30^. For example, some selection and/or additional support for study sites is needed to ensure the availability of test kits and anti-TB drugs during the trial period and some strengthening of routine data collection and recording is needed to minimize missing data. If a study is completely hands-off with regard to clinical practice it may not demonstrate effects on mortality because the system in which the test is introduced is poorly functioning. This may be useful information in that specific context (there may be little point in implementing a new diagnostic in the context of a dysfunctional health system) but may mislead on the potential impact on mortality in a better functioning system. In practice, feasibility issues such as available study funding and available time to conduct the studies ^27^ mean that most studies fall along a continuum between pragmatic and explanatory approaches ^32^. In this light, researchers are encouraged to use the Pragmatic Explanatory Continuum Indicator Summary (PRECIS) tool to inform design decisions on how explanatory (ideal context) or pragmatic (usual care context) the study features of their trials can be in the pragmatic/explanatory continuum ^33^. Trial findings also need to be interpreted in line with the trial’s position in this continuum particularly if they are labelled as pragmatic.

When a study aims to provide valid evidence for or against the introduction of a trial-validated intervention in real-world settings, a more pragmatic trial is needed to evaluate its performance in less controlled, heterogeneous settings and populations that are typical of the settings the intervention is intended for ^34^. The study population should be all persons that would qualify for the intervention under usual care including adults and children and participants that may be prone to loss to follow-up. Recruitment approaches should be built on existing ones ^35^. Individual consent should be inferred from participants’ presentation at the health facility and request for treatment especially if the intervention under study is already approved. Study populations, would, therefore present themselves to the health facility staff and be evaluated by no more effort than that observed under usual care or alternative methods of obtaining consent can be sought such as consent waivers, integrated clinical and research consent, and broadcast consent (notifications in health settings informing patients that trials with minimal risk are permitted) ^36^. The intervention should be delivered through usual care providers and resources ^34^. Data on patients and outcome measures should also be gathered from routine programmatic data sources wherever efforts can be made to strengthen data collection and bring it to a higher standard, without having the potentially problematic effect of placing research staff at each study site.

The strengths of our review include a comprehensive search in multiple databases for studies assessing the effect of Xpert MTB/RIF testing on mortality. Two reviewers extracted data in discussion with a senior reviewer. Our review was limited by focusing on the effect of Xpert MTB/RIF on one health outcome. However, other health outcomes such as morbidity and quality of life are limited by lack of standardized scores and are rarely ^18^ measured in trials. For example, only one trial ^10^ in our review evaluated the effect on morbidity and none evaluated the effect on quality of life. An advantage of Xpert is its high sensitivity in detecting rifampicin-resistant TB ^37^. It would be informative to evaluate the effect of Xpert MTB/RIF on health outcomes in patients with rifampicin-resistant TB. However, none of the included studies evaluated the effect of Xpert on rifampicin resistant-TB due to limited prevalence and follow-up. In addition, we did not review the effect of Xpert testing in children because TB diagnosis in children is still a challenge ^38^. Indeed, only one study ^11^ included children. Lastly, our review was limited to studies written in English and to what was reported in the included studies.

In conclusion, although presented as pragmatic, specific study design and execution choices are likely to reduce the ability of trials to demonstrate an impact of Xpert MTB/RIF testing on mortality. Offering higher quality of care than what occurs in usual care may lead to differences in mortality between control and intervention arms that are smaller than would have been observed with usual care. Trialists face an inherent tension between balancing internal and external validity. Nonetheless, our findings indicate trials that are further along the explanatory-pragmatic continuum are needed to evaluate the impact of the next-generation of TB diagnostics in real-world settings.

## Data availability

### Underlying data

All data underlying the results are available as part of the article and no additional source data are required.

### Extended data

Open Science Framework: Variation in the observed effect of Xpert MTB/RIF testing for tuberculosis on mortality. https://doi.org/10.17605/OSF.IO/HXYQW ^20^.

This project contains the following extended data:

- Protocol-Literature Review_v4 (protocol for this review; see https://osf.io/hxyqw/files/).
- Systematic review data-TB Xpert Effect (data on studies identified by this review; see https://osf.io/hxyqw/files/).

### Reporting guidelines

Open Science Framework: PRISMA checklist and flow chart for ‘Variation in observed effect of Xpert MTB/RIF testing for tuberculosis on mortality’ https://doi.org/10.17605/OSF.IO/HXYQW ^20^.

The PRISMA checklist is available at https://osf.io/hxyqw/files/.

Data are available under the terms of the Creative Commons Zero "No rights reserved" data waiver (CC0 1.0 Public domain dedication).
