# Supplementary material for: Variation in the observed effect of Xpert MTB/RIF testing for tuberculosis on mortality: A systematic review and analysis of trial design considerations
Source: Wellcome Open Res. 2020 Aug 17;4:173. Originally published 2019 Nov 12. [Version 2] doi: 10.12688/wellcomeopenres.15412.2 (PMC7438967; doi:10.12688/wellcomeopenres.15412.2)
Supplement: Peer review history

# Peer review: Reviewer response for version 2

**DOI:** 10.21956/wellcomeopenres.17816.r39911

**Published:** 2020-08-25

**Authors:** Norbert O Ndjeka

**Affiliations:** 1 Drug-Resistant TB, TB and HIV directorate, National Department of Health, Pretoria, South Africa

The rationale for the study and the objectives were clearly stated. This study is very important because Xpert MTB/RIF is an important investment for TB control globally. It is therefore crucial to have this issue addressed.  

 The study focused on 5 areas: study setting and context, study population, recruitment process, study procedures and follow up. It is very interesting to note the differences and similarities among the studies reviewed. Studies reviewed have been conducted in several settings including medium burden and high burden settings.

 The arguments provided are strong, compelling, and very convincing. Issues like provision of care by research staff may clearly have an effect on outcome or mortality in particular. The performance of X-ray on a routine basis may explain the difference if not done everywhere. Management of loss to follow up is another critical factor that could have an effect on the outcome.

 In conclusion, the statistical analysis provided and interpretation supports the fact that differences identified in the 5 areas considered in this investigation explain the differences evidenced in the outcomes of the various studies. Strategies proposed to improve future designs appear to be in line with the findings of the study.

Are the rationale for, and objectives of, the Systematic Review clearly stated?

Yes

Is the statistical analysis and its interpretation appropriate?

Yes

Are sufficient details of the methods and analysis provided to allow replication by others?

Yes

Are the conclusions drawn adequately supported by the results presented in the review?

Yes

Reviewer Expertise:

I am a Specialist Family Physician with an interest in Tuberculosis Control and HIV Care.

I confirm that I have read this submission and believe that I have an appropriate level of expertise to confirm that it is of an acceptable scientific standard.

# Peer review: Reviewer response for version 2

**DOI:** 10.21956/wellcomeopenres.17816.r39989

**Published:** 2020-08-19

**Authors:** Tom Boyles

**Affiliations:** 1 Anova Health Institute, Johannesburg, South Africa; 2 London School of Hygiene and Tropical Medicine, London, UK

I have no further comments to make and agree to change my recommendation to 'approved'

Are the rationale for, and objectives of, the Systematic Review clearly stated?

Yes

Is the statistical analysis and its interpretation appropriate?

Yes

Are sufficient details of the methods and analysis provided to allow replication by others?

Yes

Are the conclusions drawn adequately supported by the results presented in the review?

Partly

Reviewer Expertise:

NA

I confirm that I have read this submission and believe that I have an appropriate level of expertise to confirm that it is of an acceptable scientific standard.

# Peer review: Reviewer response for version 1

**DOI:** 10.21956/wellcomeopenres.16852.r39064

**Published:** 2020-07-09

**Authors:** Anil Pooran

**Affiliations:** 1 Centre for Lung Infection and Immunity, Division of Pulmonology, Department of Medicine, UCT Lung Institute and South African MRC/UCT Centre for the Study of Antimicrobial Resistance, University of Cape Town, Cape Town, South Africa

The authors here conducted a systematic review to investigate the effect of several trial design parameters on patient outcomes in Xpert MTB/RIF evaluation studies

 Indeed, the majority of the clinical trials reported here have demonstrated that Xpert was not as impactful on mortality and other outcomes that directly impacts patient care as previously assumed, based on initial diagnostic performance and modelling studies.

 The authors have highlighted that several of the trials had flaws in their design that would have adversely affected the performance of Xpert over the standard of care i.e. smear microscopy in most cases, including (amongst others) the  level of care received by participants and the pre-selection of participants with high suspicion of TB. The authors also provide suggestions on strategies to improve the design of these trials, so they fall more toward the pragmatic end of the continuum and subsequently, are closer to what is actually occurring.

 The author’s arguments are very compelling and convincing, but they should also consider the practicality of these suggestions and budgetary constraints of the trial itself. Implementation of more pragmatic design elements would likely be setting-specific depending on the operational state of the healthcare system. Indeed, several of the elements that the authors argue against are included to ensure the generation of valid data in the trial. For example, in some high TB burden, low income ‘real-world’ settings, LTFU atthe primary care level can be very high. Thus, trials need strict follow-up procedures by dedicated research staff for collection of data on patient outcomes. This also has cost implications - greater LTFU means that sample sizes (and thus trial costs) would need to be increased in order to maintain the expected effect. Other considerations may also be the timing of the trial – a technology such as Xpert would need to be sufficiently established within the healthcare system and enough “real world” data available before a pragmatic trial could take place. Indeed, the authors have touched upon this when they mention the balancing act of designing a trial with both internal and external validity but a bit more discussion on the topic would be helpful.

 Thank you for allowing me the opportunity to review this article

Are the rationale for, and objectives of, the Systematic Review clearly stated?

Yes

Is the statistical analysis and its interpretation appropriate?

Yes

Are sufficient details of the methods and analysis provided to allow replication by others?

Yes

Are the conclusions drawn adequately supported by the results presented in the review?

Partly

Reviewer Expertise:

Research Scientist with an interest in diagnostics, cost effectiveness and immunology of TB

I confirm that I have read this submission and believe that I have an appropriate level of expertise to confirm that it is of an acceptable scientific standard.

# Peer review: Reviewer response for version 1

**DOI:** 10.21956/wellcomeopenres.16852.r37001

**Published:** 2019-12-11

**Authors:** Tom Boyles

**Affiliations:** 1 Anova Health Institute, Johannesburg, South Africa; 2 London School of Hygiene and Tropical Medicine, London, UK

Thank you for the opportunity to review this systematic review and analysis of trial design considerations in the evaluation of Xpert MTB/RIF.

 When Xpert MTB/RIF was first reported to have much higher sensitivity than the current test (smear microscopy), it was widely assumed to be a game changer in terms of TB diagnostics. Multiple clinical trials along with meta-analyses have shown that the impact is much less than was anticipated.

 Rather than accept that Xpert MTB/RIF might not be an important addition to TB diagnostics, multiple authors have tried to explain the lack of trial efficacy by examining the trial design.

 In this review, the authors focus on 5 areas, study setting and context, population, recruitment and enrollment, study procedures and follow-up.  

 The authors make a strong argument for many of the studies having sub-optimal design in these aspects and conclude that “trialists face an inherent tension between balancing internal and external validity. Nonetheless, our findings indicate trials that are further along the explanatory-pragmatic continuum are needed to evaluate the impact of the next-generation of TB diagnostics in real-world settings.”

 This is an interesting argument as in general, when trials are less pragmatic there tends to be a larger effect size than in real world settings. The authors are arguing that in this instance, the opposite is true, and that larger effect sizes would be seen in real world settings.

 Their arguments are convincing but do not address the main reasons for the failure of these studies to show an effect on mortality. In this reviewer’s opinion, the primary reason that many of these trials failed to show an effect of Xpert MTB/RIF was fundamental design flaws in terms of the choice of the intervention arm.

 The fundamentals of determining the impact of a novel diagnostic is to perform ‘test research’ (diagnostic accuracy studies) before moving to ‘diagnostic research’ (e.g. developing algorithms or prediction rules), before finally moving to ‘diagnostic intervention research’ (randomised trials). In the case of Xpert MTB/RIF no adequate ‘diagnostic research’ was performed and so appropriate diagnostic strategies were not developed prior to designing randomised trials. The trials therefore merely tested the standard of care, which was smear microscopy and frequent empiric therapy (due to the known lack of sensitivity of smear) vs Xpert MTB/RIF as a stand alone test. An appropriate research strategy would have been to first develop a full diagnostic strategy based around Xpert MTB/RIF, which included for example, therapy for patients with a high pre-test probability of disease regardless of Xpert result, empiric therapy for patients with high pre-test probability who were unable to produce sputum, possibly with the inclusion of a trial of antibiotics and referral for CXR when the diagnosis was in doubt.

 Such an approach would have adequately compared the current standard of care with an evidence based diagnostic strategy which included Xpert MTB/RIF. In their form that these studies were done, it was in my opinion highly likely that no effect would be seen.

 Therefore, while the authors make some convincing arguments as to how these trials might have been better designed, my suggestion would be that they acknowledge that these were minor flaws in comparison to those mentioned above. I would conclude that while there is room for improvement in the 5 areas they discuss, adequate randomised trials of Xpert MTB/RIF would compare the current standard of care diagnostic strategy based around smear microscopy. with a novel diagnostic strategy which included Xpert MTB/RIF, rather than simply comparing a test with another test.

Are the rationale for, and objectives of, the Systematic Review clearly stated?

Yes

Is the statistical analysis and its interpretation appropriate?

Yes

Are sufficient details of the methods and analysis provided to allow replication by others?

Yes

Are the conclusions drawn adequately supported by the results presented in the review?

Partly

Reviewer Expertise:

Infectious diseases clinician with an interest in the appropriate evaluation of novel diagnostics, particularly for tuberculosis.

I confirm that I have read this submission and believe that I have an appropriate level of expertise to confirm that it is of an acceptable scientific standard, however I have significant reservations, as outlined above.
